# Select Phytochemicals Reduce *Campylobacter jejuni* in Postharvest Poultry and Modulate the Virulence Attributes of *C. jejuni*

**DOI:** 10.3389/fmicb.2021.725087

**Published:** 2021-08-12

**Authors:** Basanta R. Wagle, Annie M. Donoghue, Palmy R. Jesudhasan

**Affiliations:** ^1^Department of Poultry Science, University of Arkansas, Fayetteville, AR, United States; ^2^Poultry Production and Product Safety Research Unit, Agricultural Research Service, United States Department of Agriculture, Fayetteville, AR, United States

**Keywords:** *Campylobacter jejuni*, phytochemical, chill tank treatment, chicken, molecular mechanism

## Abstract

Consumption or handling of poultry and poultry products contaminated with *Campylobacter* species are a leading cause of foodborne illness in humans. Current strategies employed to reduce *Campylobacter* in live chickens provide inconsistent results indicating the need for an alternative approach. This study investigated the efficacy of phytochemicals, namely, turmeric, curcumin, allyl sulfide, garlic oil, and ginger oil, to reduce *Campylobacter jejuni* in postharvest poultry and sought to delineate the underlying mechanisms of action. Two experiments were conducted on the thigh skin of the chicken, and each experiment was repeated twice. Samples were inoculated with 50 μl (∼10^7^ CFU/sample) of *C. jejuni* strain S-8 and allowed to adhere for 30 min. Skin samples were dipped into their respective prechilled treatment solutions (0.25 and 0.5% in experiments 1 and 2, respectively) at 4°C for an hour to simulate chilling tank treatment, followed by plating to enumerate *C. jejuni* (*n* = 3 samples/treatment/trial). The mechanisms of action(s) were investigated using subinhibitory concentration (SIC) in adhesion, quorum sensing, and gene expression analyses. Adhesion assay was conducted on the monolayers of ATCC CRL-1590 chicken embryo cells challenged with *C. jejuni* and incubated in the presence or absence of phytochemicals for 1.5 h, followed by plating to enumerate adhered *C. jejuni*. The effects of phytochemicals on quorum sensing and cell viability were investigated using *Vibrio harveyi* bioluminescence and LIVE/Dead BacLight^TM^ bacterial viability assays, respectively. In addition, droplet digital PCR determined the gene expression analyses of *C. jejuni* exposed to phytochemicals. Data were analyzed by GraphPad Prism version 9. *C. jejuni* counts were reduced by 1.0–1.5 Log CFU/sample with garlic oil or ginger oil at 0.25 and 0.5% (*p* < 0.05). The selected phytochemicals (except curcumin) reduced the adhesion of *C. jejuni* to chicken embryo cells (*p* < 0.05). In addition, all the phytochemicals at SIC reduced quorum sensing of *C. jejuni* (*p* < 0.05). The cell viability test revealed that cells treated with 0.25% of phytochemicals had compromised cell membranes indicating this as a mechanism that phytochemicals use to damage/kill *C. jejuni*. This study supports that the application of phytochemicals in postharvest poultry would significantly reduce *C. jejuni* in poultry meat.

## Introduction

*Campylobacter jejuni* and *Campylobacter coli* are foodborne pathogens that cause gastroenteritis (campylobacteriosis) in humans and affect around 95 million people annually worldwide (Scallan et al., 2011; Kirk et al., 2015). In the United States alone, more than 1.3 million human campylobacteriosis cases are reported every year and leads to an economic burden of $1.9 billion (Scallan et al., 2011; Batz et al., 2014; Marder et al., 2017). About 90% of the campylobacteriosis is caused by *C. jejuni* (Cody et al., 2013). It is also reported that ∼58% of *C. jejuni* outbreaks in 2018 were implicated to poultry and poultry products [IFSAC (Interagency Food Safety Analytics Collaboration), 2020]. This pathogen primarily resides in the gastrointestinal tract of poultry and caused contamination of meat during poultry processing. Handling of uncooked meat and consumption of undercooked meat are the primary sources of human *Campylobacter* infections [Acheson and Allos, 2001; Altekruse and Tollefson, 2003; Taylor et al., 2013; IFSAC (Interagency Food Safety Analytics Collaboration), 2020].

Controlling *Campylobacter* in poultry meat and meat products would improve food safety and prevent human campylobacteriosis. The Food Safety and Inspection Service of the United States Department of Agriculture (USDA-FSIS) has approved several antimicrobial agents, including peracetic acid (PAA), to wash poultry carcasses during processing (Crim et al., 2015; USDA-FSIS, 2021). Currently, PAA is primarily used in commercial poultry processing plants in the United States. However, consumer acceptance has decreased due to limited efficacy, discoloration of meat, and concerns over residues (Bilgili et al., 1998; EFSA BIOHAZ Panel, 2014). As an alternative to conventional chemicals, phytochemicals have been extensively investigated for controlling foodborne pathogens (Friedman et al., 2002; Kovács et al., 2016; Wagle et al., 2017a,b). Phytochemicals exhibit multiple mechanisms of action(s) against pathogens thereby limiting resistance development (Borges et al., 2016). In addition, consumer interests in phytochemicals have been increasing because these compounds have been in use since ancient times as food preservatives and flavor enhancers.

The survival and virulence of *C. jejuni* are mediated through motility, adherence, quorum sensing, and formation of biofilms. The anti-*Campylobacter* activity of phytochemicals could be due to the interference in these mechanisms. Previously, researchers have tested several phytochemicals as antimicrobial wash and coating of poultry meat against *C. jejuni* and sought the mechanisms (Lu et al., 2011, 2012; Murali et al., 2012; Castillo et al., 2014; Kovács et al., 2016; Wagle et al., 2017a, 2019a,b; Shrestha et al., 2019a,b). In addition to the antimicrobial wash, chill tank treatment of carcasses with PAA is a common practice. The latter intervention could significantly reduce contamination as the meat remains in the chill tank for a longer duration (1 h) before further processing. Therefore, in this study, we investigated the application of select phytochemicals (turmeric, curcumin, allyl sulfide, garlic oil, and ginger oil) to simulate the chill tank treatment of poultry carcasses for reducing *C. jejuni*.

The objective of this study is to determine the antimicrobial activity of select phytochemicals against *C. jejuni* in postharvest poultry and delineated the underlying mechanisms of action(s). First, the antimicrobial activity was determined as a simulation of chill tank treatment of carcass. Second, we determined the subinhibitory concentration (SIC; the highest dose that does not reduce the growth of *C. jejuni*) of each phytochemical in the broth medium. Finally, the mechanistic functions of phytochemicals against *C. jejuni* were investigated using the SIC of phytochemicals in adhesion assay, quorum sensing assays, and gene expression analyses.

## Materials and Methods

### *Campylobacter jejuni* Strain and Culture Conditions

A previously isolated and well-characterized wild-type strain (S-8) of *C. jejuni* from the cecal contents of broilers was used for this study (Wagle et al., 2020b). Strain, S-8 was cultured in 10 ml of *Campylobacter* enrichment broth (CEB, International Diagnostics Group, Bury, United Kingdom) for 48 h at 42°C under microaerophilic conditions (5% O_2_, 10% CO_2_, and 85% N_2_). After the growth, the culture was centrifuged at 1,864 × *g* for 15 min and resuspended in Butterfield’s phosphate diluent (BPD, 0.625 mM potassium dihydrogen phosphate, pH 7.2) for further use in the experiment.

### Preparation of Phytochemical Solutions

We purchased all the phytochemicals from Sigma-Aldrich Co. (St. Louis, MO, United States). Since the solubility of phytochemicals varies widely, we dissolved turmeric in water, curcumin in dimethyl-sulfoxide (DMSO), and allyl sulfide, garlic oil, and ginger oil in ethanol. A 5% stock solution of turmeric and curcumin was prepared, and 10% of the stock was prepared for the rest of the phytochemicals. The stock solution was diluted to obtain a desired concentration in the subsequent experiments.

### Antimicrobial Efficacy of Phytochemicals Against *C. jejuni* on Chicken Skin Samples

A previously published method was used for determining the antimicrobial efficacy of phytochemicals against *C. jejuni* on chicken skin (Wagle et al., 2019a). Chicken thigh skin parts were obtained from the pilot processing plant of the University of Arkansas (Fayetteville, AR, United States), and skin samples of 4 cm × 4 cm were prepared aseptically. We conducted two experiments, each experiment consisting of two trials. In all the trials, we used PAA at 220 ppm as industry control. For the first experiment, 24 skin samples were randomly assigned to eight groups (baseline, controls, PAA, and 0.25% of each phytochemical; *n* = 3 samples per treatment per trial). For the second experiment, 33 skin samples were divided randomly into 11 groups (baseline, controls, PAA, 0.5% of each phytochemical, and three combinations of ginger and garlic oil at 0.125, 0.25, and 0.5% doses; *n* = 3 samples per treatment per trial). Individual skin samples were inoculated with 50 μl of *C. jejuni* S-8 strain (∼7.2 and 7.6 Log CFU/sample in the first and second experiments, respectively). After inoculation, the samples were incubated for 30 min to facilitate adherence. The inoculated chicken skin samples were immersed in the respective treatment solutions (prechilled at 4°C) under refrigerated temperature for an hour to simulate the chill tank treatment procedure of a commercial poultry processing plant. After storage, the samples were transferred to 10 ml of Dey-Engley neutralizing broth (Difco Laboratories, Sparks, MD, United States) and vigorously vortexed for 30 s. The samples were serially diluted (1:10) and plated on *Campylobacter* Line Agar (CLA; Line, 2001) plates. The plates were incubated at 42°C for 48 h under microaerophilic conditions for *C. jejuni* enumeration.

### Determination of SIC of Phytochemicals

The SIC of phytochemicals against *C. jejuni* was determined as described previously by our laboratory (Wagle et al., 2017b, 2020a). Briefly, twofold dilutions of phytochemicals (0, 2.5, 1.25, 0.625, 0.3125, 0.1562, 0.0781, 0.0391, and 0.0195%) were made in a sterile 96-well polystyrene plate (Costar, Corning, NY, United States) containing CEB in equal volume (100 μl) followed by inoculation with *C. jejuni* S-8 strain (∼10^6^ CFU/ml). After the incubation of *C. jejuni* with phytochemicals for 24 h, the samples were diluted and plated onto CLA plates. The plates were enumerated for *C. jejuni* after incubation at 42°C under microaerophilic conditions for 48 h. The highest concentration of phytochemicals that did not inhibit the growth of *C. jejuni* as compared with controls was selected as the SIC of the compound.

### Effect of Phytochemicals on the Viability of Chicken Embryo Cells

ATCC CRL-1590 chicken embryo cells were grown and maintained in Dulbecco’s modified Eagle’s medium (DMEM; VWR Life Science, NY, United States) containing 10% heat-inactivated fetal bovine serum (Thermo Fisher Scientific, Carlsbad, CA, United States) and 5% tryptose phosphate broth (Sigma-Aldrich). The effect of phytochemicals on the viability of chicken embryo cells was determined using alamarBlue assay as described previously (Wagle et al., 2020a). Briefly, cells were cultured in 96-well tissue culture plates (Costar) at ∼10^5^ cells per well to form a monolayer at 37°C in a humidified, 5% CO_2_ incubator for 24 h. The monolayer was washed three times and treated with DMSO in DMEM, or ethanol in DMEM, or phytochemicals at SIC in DMEM or DMEM (control) for 2 h. Negative controls without chicken embryo cells containing DMEM were also included. AlamarBlue reagent was added to each well, including blanks and negative controls, and incubated at 37°C for 1 h. The fluorescence was read before and after incubation using Cytation 5 multimode reader (BioTek Instruments, Inc., Winooski, VT, United States) at 560/590 nm (excitation/emission).

### Effect of Phytochemicals on Adhesion of *C. jejuni* to Chicken Embryo Cells

The effect of phytochemicals on adhesion of *C. jejuni* S-8 to ATCC CRL-1590 chicken embryo cells was investigated as previously reported (Koo et al., 2012; Wagle et al., 2020a). Briefly, chicken embryo cells were grown and maintained as described above to form a monolayer. Chicken embryo cell monolayer was inoculated with mid-log phase (10 h) of *C. jejuni* S-8 (∼6 Log CFU/well; multiplicity of infection, 10:1) either with DMEM alone (control) or in the presence of DMSO, ethanol, or SIC of phytochemicals. The inoculated embryo cells were incubated at 42°C for 1.5 h in a microaerophilic environment. Thereafter, the inoculated monolayers were rinsed three times in minimal media, followed by lysis with 0.1% Triton X-100 (Invitrogen, Carlsbad, CA, United States) for 15 min. The number of adhered *C. jejuni* was determined by serial dilution and plating of embryo cells lysate on CLA plates. The plates were incubated under microaerophilic conditions at 42°C for 48 h.

### Effect of Phytochemicals on Quorum Sensing Activity of *C. jejuni*

The effect of SIC of phytochemicals on autoinducer-2 (AI-2) levels of *C. jejuni* S-8 was determined using *Vibrio harveyi* bioluminescence assay as described previously (Bassler et al., 1994; Castillo et al., 2014; Wagle et al., 2020a). *C. jejuni* S-8 in the presence or absence of SIC of phytochemicals was cultured to mid-log phase and centrifuged at 1,864 × *g* for 10 min. The supernatant was collected and filtered using a 0.2-μm syringe filter to obtain the cell-free supernatant (CFS). Likewise, *V. harveyi* strain BB152 was grown overnight in Luria Bertani broth (HiMedia Laboratories Pvt. Ltd., Mumbai, India) at 30°C, and the CFS was prepared. The reporter strain, *V. harveyi* (BB170), was also cultured in Luria Bertani broth at 30°C for 24 h. The culture was diluted 1:5,000 with autoinducer assay medium (Inoculum ∼ 3.5 Log CFU/ml), and 90 μl of the diluted culture was dispensed into 96-well microtiter plates. Ten microliters of CFS of *V. harveyi* BB152 (positive control) or CFS of phytochemical-treated or untreated *C. jejuni* or autoinducer assay medium (negative control) was added in triplicates in the plate. The mixture was well mixed, and the luminescence was measured at 30°C at 20 min intervals for 8 h using Cytation 5 multimode reader. The luminescence observed in the negative controls due to the self-induction of *V. harveyi* (BB170) was deducted from the positive controls and treatments before further analysis.

### Effect of Phytochemicals on the Viability of *C. jejuni*

We determined the effect of phytochemicals on the viability of *C. jejuni* S-8 using a Live/Dead BacLightTM bacterial viability kit (Thermo Fisher Scientific) as per the manufacturer’s protocol. Briefly, *C. jejuni* S-8 was cultured overnight at 42°C under microaerophilic conditions. The culture was dispensed in the 24-well plates and treated with 0.25% of the phytochemicals for 5 min, followed by the addition of SYTO-9 and propidium iodide. SYTO-9 and propidium iodide stains were used for the differential staining of live and dead cells. The cells were transferred to the microscope slide and observed under the fluorescent microscope (BZ-810, Keyence Co., IL, United States) at a ×20 objective lens.

### Effect of Phytochemicals on the Gene Expression of *C. jejuni*

The effect of phytochemicals on the expression of *C. jejuni* S-8 genes critical for survival and virulence was studied using digital droplet PCR (ddPCR) as described previously with slight modifications (Woodall et al., 2005; Wagle et al., 2017b). *C. jejuni* strain S-8 was grown in the presence or absence of SIC of phytochemicals in CEB at 42°C under microaerophilic conditions till the mid-log phase. The total RNA was extracted using RNA mini kit (Invitrogen) and treated with DNase (Thermo Fisher Scientific). The complementary DNA (cDNA) was prepared using an iScript cDNA synthesis kit (Bio-Rad). All the primers in our study (Table 1) were designed from published GenBank *C. jejuni* sequences using Primer 3 software (National Center for Biotechnology Information) and obtained from Integrated DNA Technologies. Twenty microliters of the reaction mixture were prepared by adding EvaGreen supermix (Bio-Rad), forward and reverse primers, and cDNA. Assay controls containing only the primes and EvaGreen supermix were also prepared. The droplets were made as per the manufacturer’s protocol in QX200^TM^ droplet generator (Bio-Rad) and subjected to PCR in a thermal cycler (C1000, Bio-Rad). The plate was read using QX200^TM^ droplet reader. Data were normalized to an endogenous control (16S rRNA) by calculating the ratio of copies of the target gene to the reference gene for the control and treatments before further analysis.

**TABLE 1:** Primers used for gene expression analysis using digital droplet PCR.

Gene with Accession No.	Primer	Sequence (5′-3′)
16S-rRNA (NC_002163.1) (product length 78 bp)	Forward Reverse	5′-ATAAGCACCGGCTAACTCCG-3′ 5′-TTACGCCCAGTGATTCCGAG-3′
*motA* (NC_002163.1) (product length 75 bp)	Forward Reverse	5′-AGCGGGTATTTCAGGTGCTT-3′ 5′-CCCCAAGGAGCAAAAAGTGC-3′
*motB* (NC_002163.1) (product length 51 bp)	Forward Reverse	5′-AATGCCCAGAATGTCCAGCA-3′ 5′-AGTCTGCATAAGGCACAGCC-3′
*cetA* (NC_002163.1) (product length 78 bp)	Forward Reverse	5′-CCTACCATGCTCTCCTGCAC -3′ 5′-CGCGATATAGCCGATCAAACC-3′
*cadF* (NC_002163.1) (product length 135 bp)	Forward Reverse	5′-CGCGGGTGTAAAATTCCGTC-3′ 5′-TCCTTTTTGCCACCAAAACCA-3′
*ciaB* (NC_002163.1) (product length 50 bp)	Forward Reverse	5′-TCTCAGCTCAAGTCGTTCCA-3′ 5′-GCCCGCCTTAGAACTTACAA-3′
*jlpA* (NC_002163.1) (product length 66 bp)	Forward Reverse	5′-AGCACACAGGGAATCGACAG-3′ 5′-TAACGCTTCTGTGGCGTCTT-3′
*sodB* (NC_002163.1) (product length 98 bp)	Forward Reverse	5′-CAAAACTTCAAATGGGGGCGT-3′ 5′-CACAGCCACAGCCTGTACTT -3′
*katA* (NC_002163.1) (product length 99 bp)	Forward Reverse	5′-ATGCTGAACGCGATGTGAGA-3′ 5′-CGCGGATGAAGAATGTCGGA-3′
*luxS* (NC_002163.1) (product length 106 bp)	Forward Reverse	5′-AGTGTTGCAAAAGCTTGGGA-3′ 5′-GCATTGCACAAGTTCCGCAT-3′

### Statistical Analyses

*Campylobacter jejuni* counts were logarithmically transformed before analysis to achieve homogeneity of variance (Byrd et al., 2001). All experiments were completely randomized design and had six replicates except for the gene expression analysis, which had three biological replicates. The data from independent trials were pooled and analyzed using ANOVA on GraphPad Prism version 9.1 (GraphPad Software, San Diego, CA, United States). Tukey’s test for multiple comparisons among treatments was used in all the assays except for the quorum sensing assay, where Sidak’s multiple comparisons test was used. A *p*-value of < 0.05 was considered statistically significant.

## Results

### Antimicrobial Efficacy of Phytochemicals Against *C. jejuni* on Chicken Skin Samples

**FIGURE 1:**
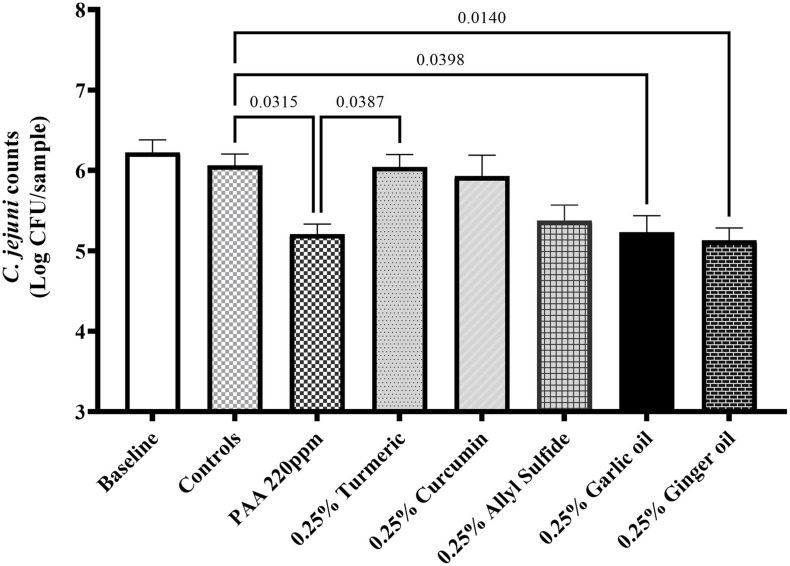
Antimicrobial efficacy of phytochemicals as chill tank treatment at 0.25% dose in reducing *Campylobacter jejuni* strain S-8 on chicken skin. Chicken skin samples were inoculated with 7.2 Log CFU/sample of *C. jejuni*. Results are averages of two independent trials, each containing triplicate samples (mean and SEM). The estimated *p*-values < 0.05 are included above the bars.

**FIGURE 2:**
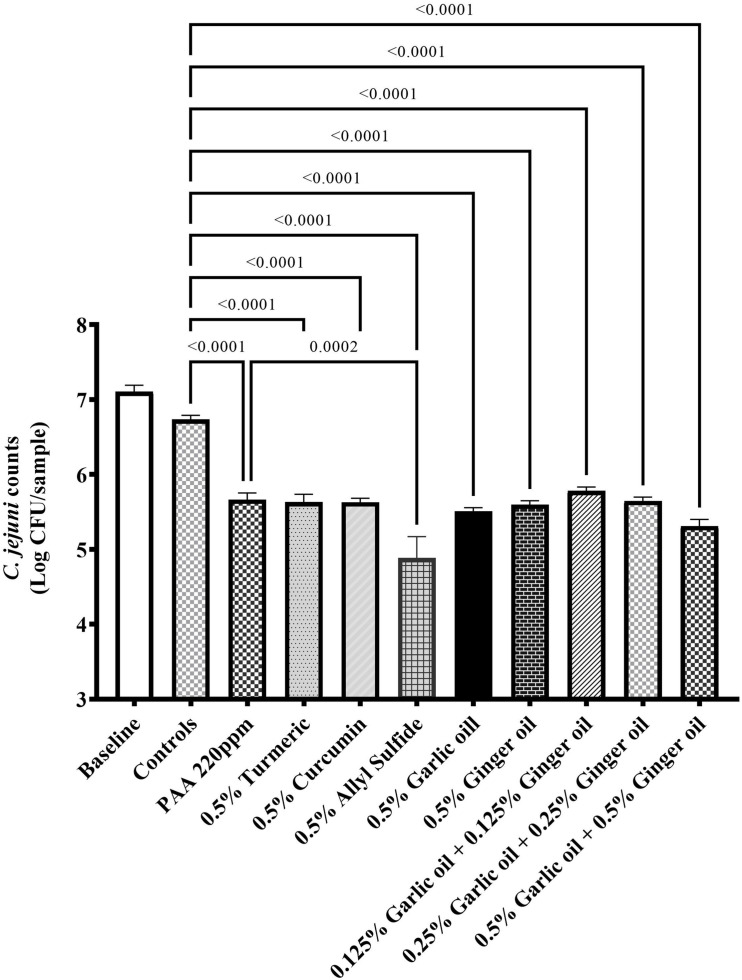
Antimicrobial efficacy of phytochemicals as chill tank treatment at 0.5% dose and three combinations of garlic oil and ginger oil in reducing *C. jejuni* strain S-8 on chicken skin. Chicken skin samples were inoculated with 7.6 Log CFU/sample of *C. jejuni*. Results are averages of two independent trials, each containing triplicate samples (mean and SEM). The estimated *p*-values < 0.05 are included above the bars.

The effect of phytochemicals against *C. jejuni* on chicken skin is presented in Figures 1,2. In the first experiment, the skin samples in the controls (dipped in BPD) had ∼6 Log CFU/sample of *C. jejuni* surviving on the surface (Figure 1). There was a reduction of ∼0.85 Log CFU/sample with PAA at 220 ppm. Garlic oil and ginger oil at 0.25% reduced the counts by ∼0.83 and 0.93 Log CFU/sample, respectively, compared with controls. The garlic oil and ginger oil were as effective as PAA in reducing *C. jejuni*. In contrast to garlic oil and ginger oil, turmeric, curcumin, and allyl sulfide at 0.25% dose failed to reduce *C. jejuni* counts compared with the controls. In the second experiment, the control samples had ∼6.7 Log CFU/sample of *C. jejuni* (Figure 2). Similar to experiment 1, PAA at 220 ppm significantly reduced *C. jejuni* counts by 1 Log CFU/sample. In contrast to the effect of 0.25% in experiment 1, turmeric, curcumin, and allyl sulfide at 0.5% significantly reduced *C. jejuni* counts compared with control samples, and these phytochemicals were as effective as PAA. The 0.5% of garlic oil and ginger and their three combinations at 0.125, 0.25, and 0.5% doses also produced significant reductions. However, the combination of treatments did not provide an additional reduction in *C. jejuni* counts compared with their respective individual doses.

### Determination of SIC of Phytochemicals

**FIGURE 3:**
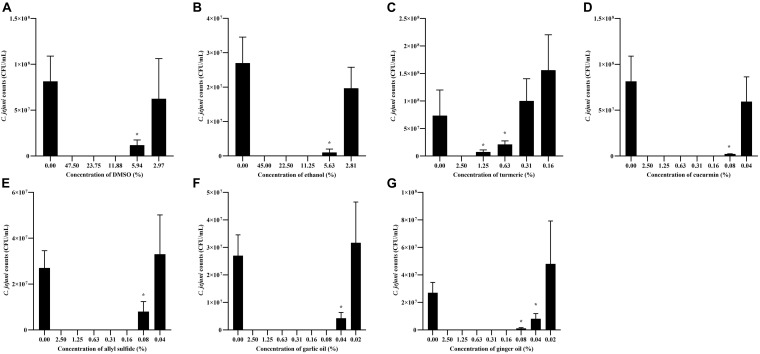
Determination of SIC of DMSO **(A)**, ethanol **(B)**, turmeric **(C)**, curcumin **(D)**, allyl sulfide **(E)**, garlic oil **(F)**, and ginger oil **(G)** against *C. jejuni* S-8 in a microtiter plate. Results are averages of three independent experiments, each containing duplicate samples (mean and SEM). *Indicates a significant difference between control and treatment.

The effect of different concentrations of phytochemicals on the growth of *C. jejuni* S-8 is shown in Figure 3. The highest concentration of turmeric that did not significantly inhibit the growth of *C. jejuni* S-8 was 0.31%, which was selected as the SIC of turmeric (Figure 3C). Similarly, curcumin at 0.04% did not affect the growth of *C. jejuni* and at this dose has 0.75% of diluent (DMSO) (Figure 3D). The DMSO failed to reduce the growth of *C. jejuni* even at the higher concentration (2.97%) (Figure 3A). Likewise, the SICs of allyl sulfide, garlic oil, and ginger oil were 0.04, 0.02, and 0.02%, respectively (Figures 3E–G). The concentration of ethanol in the 0.04% allyl sulfide is 0.36%, and this concentration did not affect the growth of *C. jejuni* S-8 (Figure 3B). These SICs of phytochemicals were used for subsequent mechanistic analysis with 0.75% DMSO and 0.36% ethanol.

### Effect of Phytochemicals on the Viability of Chicken Embryo Cells

**FIGURE 4:**
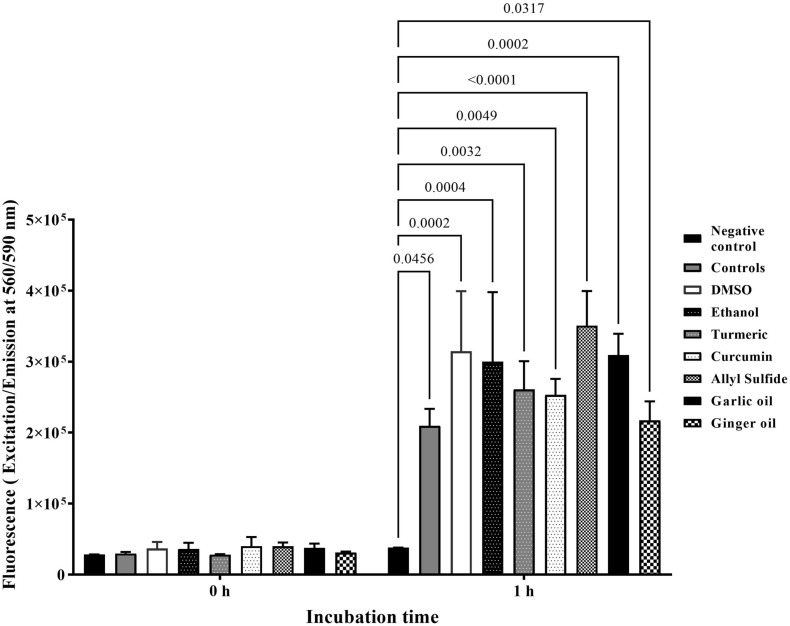
Effect of SIC of phytochemicals on viability and proliferation of ATCC CRL-1590 chicken embryo cells determined using alamarBlue Cell Viability test. Chicken embryo cells were incubated in the presence or absence of phytochemicals for 1 h, and fluorescence was measured. Results are averages of two independent experiments, each containing triplicate samples (mean and SEM). The estimated *p*-values < 0.05 are included above the bars.

Figure 4 shows the effect of phytochemicals at SIC on the viability of chicken embryo cells. There were no significant differences among negative controls, other controls (containing cells), and treatments immediately after the addition of alamarBlue reagents (0 h). However, the fluorescence level was significantly increased by at least 10 times in the controls (2 × 10^5^) compared with negative controls (<2 × 10^4^) after 1 h of incubation, indicating that the cells were viable and proliferating. In addition, there was no significant difference between controls and phytochemical-treated chicken embryo cells.

### Effect of Phytochemicals on Adhesion of *C. jejuni* to Chicken Embryo Cells

**FIGURE 5:**
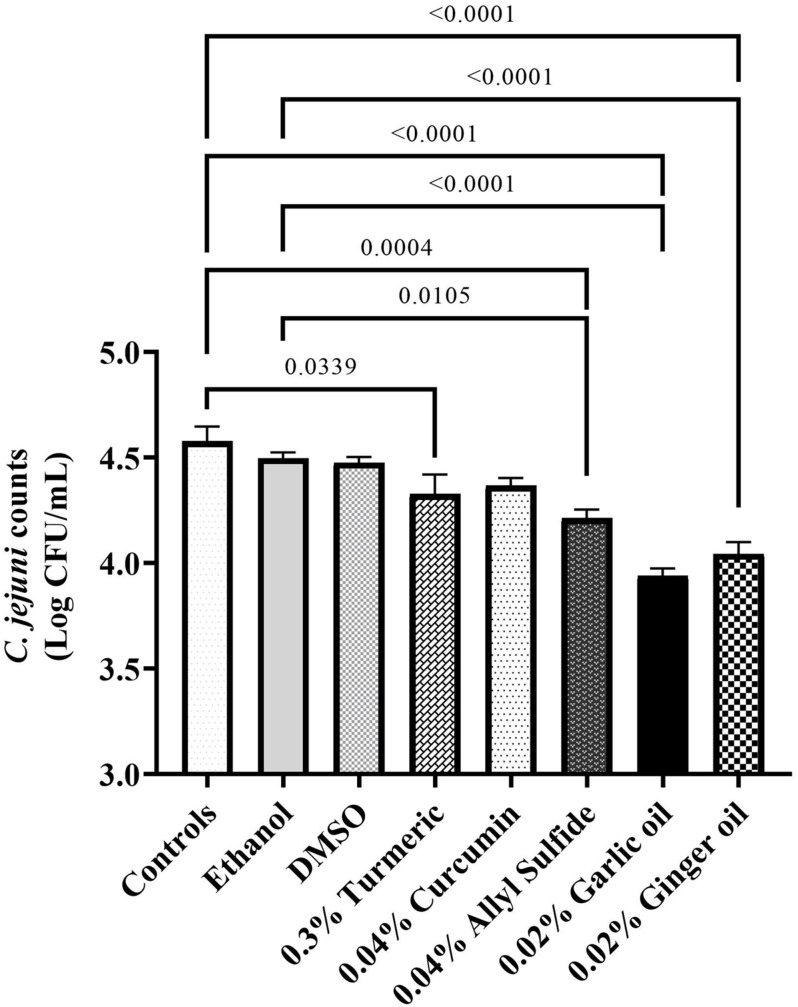
Effect of dimethyl-sulfoxide (DMSO), ethanol, turmeric, curcumin, allyl sulfide, garlic oil, and ginger oil at SIC dose on the attachment of *C. jejuni* wild-type S-8 to ATCC CRL-1590 chicken embryo cells. Results are averages of two independent experiments, each containing triplicate samples (mean and SEM). The estimated *p*-values < 0.05 are included above the bars.

Figure 5 shows the effect of phytochemicals at SIC levels on *C. jejuni* S-8 attachment to chicken embryo cells. An approximately 4.5 Log CFU/ml of *C. jejuni* S-8 adhered to the chicken embryo cells without phytochemicals (controls). Compared with controls, the SICs of turmeric and allyl sulfide decreased attachment of *C. jejuni* S-8 by ∼0.25 Log CFU/ml (*p* < 0.05). The garlic oil and ginger oil at SIC were most effective in reducing the adhesion of *C. jejuni* (∼0.63 and 0.53 Log reductions, respectively) to chicken embryo cells. However, the presence of curcumin at 0.04%, ethanol, and DMSO did not affect the attachment of *C. jejuni*.

### Effect of Phytochemicals on Quorum Sensing (AI-2) Activity of *C. jejuni*

**FIGURE 6:**
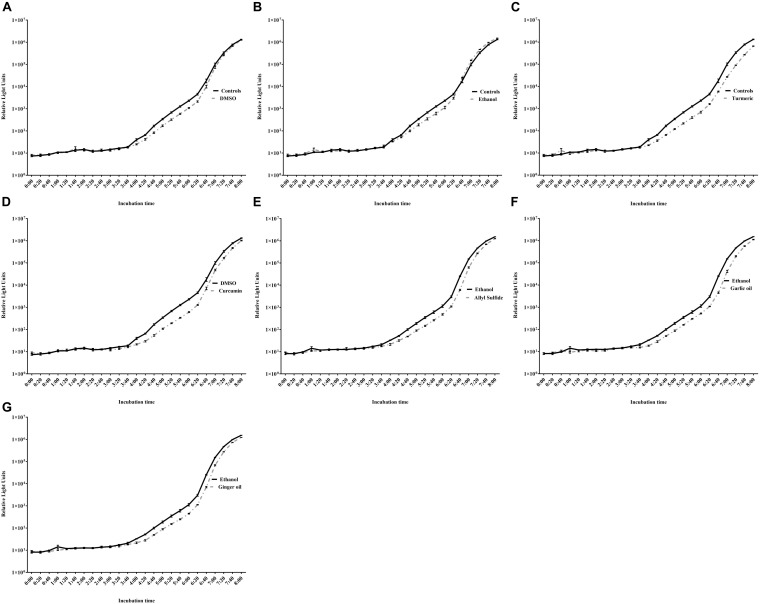
Effect of DMSO **(A)**, ethanol **(B)**, turmeric **(C)**, curcumin **(D)**, allyl sulfide **(E)**, garlic oil **(F)**, and ginger oil **(G)** at SIC dose on AI-2 levels of *C. jejuni* S-8 determined by bioluminescence assay. Results are averages of two independent experiments, each containing triplicate samples (mean and SEM).

The effect of SIC of phytochemicals on AI-2 levels of *C. jejuni* S-8 is shown in Figure 6. The AI-2 levels were ∼10^6^ relative light units (RLU) in the controls (absence of phytochemicals) at the end of 8 h. The presence of SIC of turmeric reduced AI-2 levels in *C. jejuni* S-8 from 4:20 h after incubation and maintained reduction till the end of 8 h (reduction of 6.65 × 10^5^ RLU) as compared with controls (*p* < 0.05) (Figure 6C). Similarly, 0.04% curcumin significantly reduced AI-2 levels by ∼2.3 × 10^5^ RLU at the end of 8 h compared with its control (DMSO; Figure 6D). The SIC of allyl sulfide, garlic oil, and ginger oil effectively reduced the production of AI-2 in *C. jejuni* S-8 starting from 4 h after incubation (Figures 6E–G). DMSO and ethanol also had similar effects between 4:20 and 6:20 h; however, they failed to reduce the production of AI-2 levels from 6:20 to 8 h (Figures 6A,B).

### Effect of Phytochemicals on the Viability of *C. jejuni*

**FIGURE 7:**
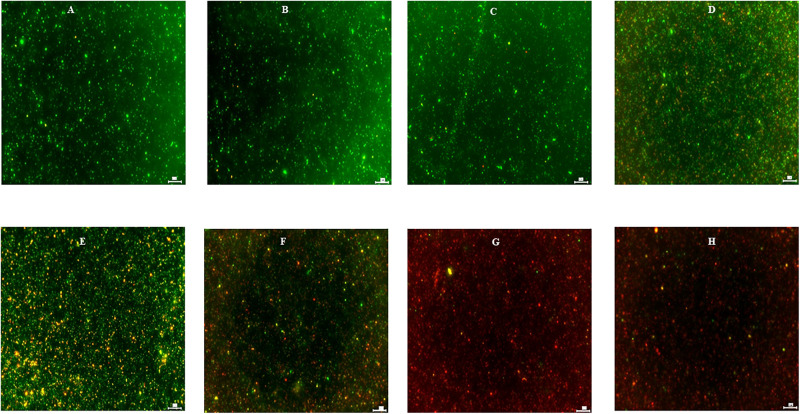
Effect of DMSO **(B)**, ethanol **(C)**, turmeric **(D)**, curcumin **(E)**, allyl sulfide **(F)**, garlic oil **(G)**, and ginger oil **(H)** on viability of *C. jejuni* S-8 cells determined by Live/Dead BacLight^TM^ bacterial viability stain under fluorescent microscope at ×20. Cells were exposed to 0.25% of phytochemicals or buffer [control; **(A)**] for 5 min at room temperature before examination. Scale bar is 50 μm.

The effect of turmeric, curcumin, allyl sulfide, garlic oil and ginger oil on the viability of *C. jejuni* S-8 was visualized using the fluorescent microscope after staining (respectively in Figures 7D–H). In the controls, most *C. jejuni* cells were live (stained green; Figure 7A); however, cells were dead (stained red) after treatments with 0.25% phytochemicals. The garlic oil and ginger oil were equally effective in killing *C. jejuni* and were more efficacious than turmeric, curcumin and allyl sulfide. The majority of *C. jejuni* cells were not affected after treatment with DMSO and ethanol (Figures 7B,C, respectively).

### Effect of Phytochemicals on the Gene Expression of *C. jejuni*

**FIGURE 8:**
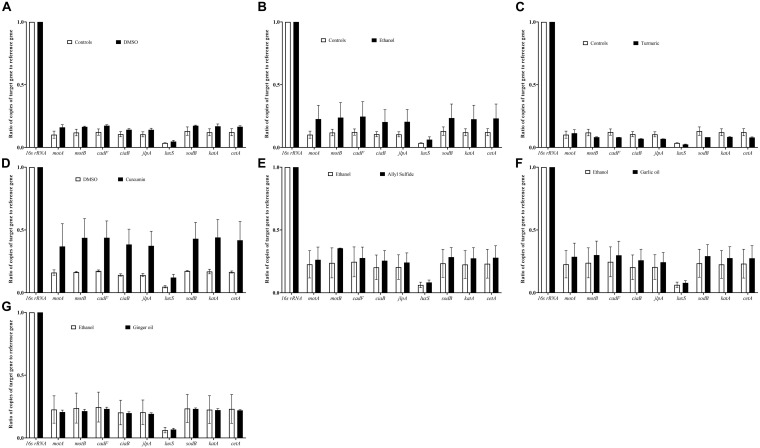
Effect of DMSO **(A)**, ethanol **(B)**, turmeric **(C)**, curcumin **(D)**, allyl sulfide **(E)**, garlic oil **(F)**, and ginger oil **(G)** at SIC dose on expression of *C. jejuni* S-8 genes critical for survival and virulence. *C. jejuni* (∼6.0 Log CFU/ml) in the presence of SIC of phytochemicals was incubated at 42°C for 12 h followed by RNA extraction and complementary DNA (cDNA) synthesis. Digital droplet PCR (ddPCR) was conducted with 16S-rRNA serving as endogenous control. Results are averages of three biological replications (mean and SEM).

Figure 8 shows the effect of SIC of phytochemicals on the expression of *C. jejuni* genes critical for virulence and survival in the meat environment. The SIC of turmeric reduced the expression of the majority of the genes tested; however, the reduction was not significant. In contrast to turmeric, curcumin at 0.04% upregulated the expression without considerable change. Similarly, we did not observe any change in the expression of *C. jejuni* S-8 genes upon exposure to SIC of allyl sulfide, garlic oil, and ginger oil and with controls, DMSO, and ethanol as well.

## Discussion

Contamination of poultry meat with foodborne pathogens is one of the major sources of bacterial gastroenteritis in humans in the United States (Mangen et al., 2016; Marder et al., 2017). To limit human exposure to *C. jejuni via* poultry meat and products, the USDA-FSIS enacted a rule in 2015 requiring additional performance standards for testing of *Campylobacter* on raw chickens, thereby reducing 33% of related human illness by 2020 (USDA FSIS, 2015). Yet, the infection caused by *Campylobacter* significantly increased by ∼13% in 2019 compared with the data of 2016–2018 (Tack et al., 2020). Therefore, the multihurdle interventions from farm to fork are utmost for pathogen reduction to a safer level in the poultry meat. In this regard, we previously investigated the potential use of phytochemicals in preharvest chickens (as feed and water additives) and on the postharvest poultry (as an antimicrobial wash treatment of carcass) for reducing *C. jejuni* (Wagle et al., 2017a,b, 2019a,b; Shrestha et al., 2019a,b). Since these results were promising, an additional application of phytochemical representing a chill tank treatment of commercial poultry processing step has been undertaken in the current research.

Antimicrobial wash treatment studies using conventional chemicals (such as chlorine, PAA, sodium hydroxide, sodium carbonate) have been extensively investigated on postharvest poultry (Zhao and Doyle, 2006; Bauermeister et al., 2008; Birk et al., 2010; Kim et al., 2017). However, limited studies have been conducted utilizing the phytochemical treatment with exposure time (1 h) and temperature (4°C) reflecting the chill tank treatments of poultry processing. In addition, phytochemicals are deemed safe, effective, and environmentally friendly compared with conventional chemicals. The reductions obtained with garlic oil and ginger oil at 0.25% and turmeric, curcumin, and allyl sulfide at 0.5% were similar to PAA at 220 ppm (Figures 1,2). The tested PAA concentration was higher than the recommended concentration (10–60 ppm for up to 120 min exposure) of PAA in the main chiller for poultry processing (EnviroTech FDA FCN, 2020). Therefore, this indicated that the phytochemicals have great potential for reducing contamination of poultry meat with *C. jejuni* S-8. Our findings aligned with Murali et al. (2012), who reported that a 10% turmeric as a marinade of chicken breast significantly reduced *C. jejuni* (∼1 Log CFU) within an hour of exposure. In contrast to our study, 2–3 Log CFU reductions of *C. jejuni* were reported with 0.25% garlic oil against *C. jejuni* in broth media at 4°C (Lu et al., 2011). In addition, they reported that the antimicrobial efficacy of phytochemicals increased with higher temperatures (22 and 35°C). The higher reductions in their study could be due to the absence of meat environment (especially fats) in the broth media. We have also included active compounds of turmeric and garlic oil (curcumin and allyl sulfide, respectively) in determining if their active compounds could be more efficacious. However, active components did not provide additional reductions of *C. jejuni*. In addition, neither increasing dose of garlic oil and ginger oil from 0.25 to 0.5% nor their combinations provide any further reduction. Similar findings were reported previously with eugenol and carvacrol as an antimicrobial wash treatment of *C. jejuni* on postharvest poultry (Shrestha et al., 2019a; Wagle et al., 2019a). This could be due to the hiding of bacteria in the crevices and empty feather follicles as *C. jejuni* is well known to reside in the pores of chicken skin (Chantarapanont et al., 2003; Jang et al., 2007), so sufficient concentrations could not reach. Therefore, novel carrier methods for delivering the phytochemicals to inactive the pathogen in these sites should be investigated in the future.

To determine the underlying mechanism of action(s) of phytochemicals, we used SIC dose as this dose is known to modulate the virulence attributes of pathogens without killing them (Upadhyay et al., 2017; Wagle et al., 2017b, 2019a,b, 2020a). Adhesion of *C. jejuni* to the chicken skin surfaces is critical for survival and virulence in the meat environment. Since chicken skin epithelial cell lines are not commercially available, we used chicken embryo cells for adhesion assays. The invasion assay was not conducted as *C. jejuni* is less likely to invade the chicken epithelial cells (Hermans et al., 2011). The tested phytochemicals (turmeric, allyl sulfide, garlic oil, and ginger oil) affected the adhesion of *C. jejuni* to chicken embryo cells (Figure 5) without affecting their viability and proliferation (Figure 4). Bensch et al. (2011) had also reported the antiadhesive activity of ginger oil and garlic oil against *C. jejuni* NCTC 81-176 on HT-29 (Human colon adenocarcinoma) cells. Similar results were reported in *C. jejuni* with berries (Salaheen et al., 2014), carvacrol (Wagle et al., 2020a), cranberry extract (Ramirez-Hernandez et al., 2015), *Alpinia katsumadai* extracts (Šikiæ Pogaèar et al., 2015), grape extract (Klanènik et al., 2017), thyme extracts (Šikiæ Pogaèar et al., 2016), herbal extracts (Bensch et al., 2011), β-resorcylic acid (Wagle et al., 2017a), and resveratrol (Klanènik et al., 2017). Thus, the observed reduction in *C. jejuni* counts on chicken skin could be due to the antiadhesion properties of phytochemicals (Figures 1,2).

Quorum sensing in bacteria including *C. jejuni* is essential for motility, biofilm formation, colonization, and interaction with epithelial cells. Quorum sensing in *C. jejuni* is mediated by producing an AI-2 molecule from S-ribosylhomocysteine (Bassler et al., 1994; Castillo et al., 2014). The *luxS* gene is responsible for cleaving S-ribosylhomocysteine into homocysteine and 4,5-dihydroxy-2,3-pentanedione (DPD). The DPD undergoes spontaneous cyclization to form AI-2 (Plummer, 2012). The AI-2 levels were significantly reduced in the presence of SIC of all the tested phytochemicals (Figure 6). Similar findings were reported with carvacrol (Wagle et al., 2020a), citrus extracts (Castillo et al., 2014), and *Euodia ruticarpa* (Bezek et al., 2016) against *C. jejuni*. These reports indicated that the interactions of *C. jejuni* with epithelial cells could be reduced due to compromised quorum sensing after phytochemical treatments.

To determine whether the phytochemicals affect the cell membrane of the pathogens thereby killing them, we visualized phytochemical-treated and un-treated *C. jejuni* after differential staining (Figure 7). The majority of the phytochemical-treated *C. jejuni* were stained red indicating that the phytochemical disrupts the cell membrane. However, whether there is a direct or indirect effect of phytochemicals on the cell membrane is unknown. Previously, Lu et al. (2012) had also reported disruption in cell membrane integrity of *C. jejuni* after treatment with allyl sulfide. Other phytochemicals such as *trans*-cinnamaldehyde, eugenol, carvacrol also disrupt the cell membrane integrity of various pathogens including *C. jejuni* (Upadhyay et al., 2013; Wagle et al., 2019c). The garlic oil and ginger oil effectively killed *C. jejuni* than turmeric, curcumin, and allyl sulfide. The killing could be due to higher antibacterial activities, as revealed by SIC doses (Figure 3). The garlic oil and ginger oil had lower SIC (0.02%). In contrast, turmeric, curcumin, and allyl sulfide had higher SIC doses (0.31, 0.04, and 0.04%, respectively). The better effectiveness of garlic oil than its active component (allyl sulfide) is also due to the presence of other active compounds such as allicin, vinyl dithiin, ajoene, and diallyl polysulfides. These active components are also known to have antimicrobial activity against various pathogens, including *C. jejuni* (Robyn et al., 2013; Nakamoto et al., 2020).

The survivability and virulence of *C. jejuni* in the meat are contributed by various genes including motility genes (*motA*, *motB*), energy taxis gene (*cetA*), attachment genes (*jlpA*, *cadF*, *ciaB*), quorum sensing gene (*luxS*), and stress-response genes (*sodB*, *katA*). Previously, it was reported that motility genes contribute to *C. jejuni* movement toward the substrate and subsequent survival at 4°C (Hazeleger et al., 1998). Besides *motA* and *motB*, the energy taxis gene (cetA) is also responsible for movement in response to the substrate (Kalmokoff et al., 2006). In addition, *cadF* and *jlpA* are responsible for cell surface attachment (Jin et al., 2003; Hermans et al., 2011). The *luxS* gene is critical for quorum sensing in *C. jejuni* and survival in the meat environment. It has been reported previously that *C. jejuni luxS* mutants were unable to survive in the chicken meat juice (Ligowska et al., 2011). The adaption of *C. jejuni* to the new environment in the meat and its survival is mediated by the stress response (*katA*, *sodB*) genes (Atack and Kelly, 2009). Therefore, we selected these genes for gene expression analysis of *C. jejuni* in response to phytochemical treatments to delineate the potential mechanism of action. We did not observe significant modulation of *C. jejuni* genes after exposure to SIC of phytochemicals (Figure 8). Although the expression of *luxS* was not significantly reduced, the productions of AI-2 was decreased (Figure 6), indicating that the phytochemical could affect the process of AI-2 production from *S*-ribosylhomocysteine. These phytochemicals could affect other genes (such as *fliA fliB* for motility, and *ahpC* for stress regulation) in *C. jejuni*. Therefore, a transcriptomics study of the *C. jejuni* after exposure to phytochemicals is warranted for an in-depth understanding of the gene expression profile.

In conclusion, the tested phytochemicals reduced *C. jejuni* counts on chicken skin. The anti-*Campylobacter* action of these phytochemicals could be mediated via the reduced adherence, decreased production of quorum sensing molecule, and disruption of cell surface structure of pathogen. Further research on the effect of phytochemicals on the sensory quality of the treated meat is warranted.

## Data Availability Statement

The raw data supporting the conclusions of this article will be made available by the authors, without undue reservation.

## Author Contributions

BW and PJ designed the study. BW conducted the experiments and wrote the manuscript. AD and PJ critically analyzed and revised the manuscript. All authors contributed to the article and approved the submitted version.

## Author Disclaimer

Mention of a trade name, proprietary product, or specific equipment does not constitute a guarantee or warranty by the USDA and does not imply its approval to the exclusion of other products that may be suitable

## Conflict of Interest

The authors declare that the research was conducted in the absence of any commercial or financial relationships that could be construed as a potential conflict of interest.

## Publisher’s Note

All claims expressed in this article are solely those of the authors and do not necessarily represent those of their affiliated organizations, or those of the publisher, the editors and the reviewers. Any product that may be evaluated in this article, or claim that may be made by its manufacturer, is not guaranteed or endorsed by the publisher.
